# 

**Published:** 1857-12

**Authors:** 


### CONTENTS OF THE DECEMBER NUMBER.

### ORIGINAL COMMUNICATIONS.

Page.

ART. I.—Fractures of the Inferior Maxilla. By Frank H. Hamilton, M, D., - - - - - 385

ART. II.—Reports of Cases occurring in the Medical Ward of the Buffalo Hospital. Reported by Dr. A. W. Nichols, - - - - - - - - - - - - - - - - - - - 398

ART. III.—Abstract of the Proceedings of the Buffalo Medical Association, - - - - - - 403

ART. IV.—Human Histology, in its Relations to Descriptive Anatomy, Physiology, and Pathology, 405

ART. V.—Monthly Record of Deaths in the City of Buffalo. By C. L. Dayton, M.D., Health Physician, - - - - - - - - - - - - - - - - - - - 414

### ECLECTIC DEPARTMENT.

The Death of Charlotte Bronte, - - - - - - - - - - - - - - - 416

The Duties of State Assayers in Relation to Quack Medicines, - - - - - - - - 417

Large Anæurismal Tumor successfully Treated by a New Method, - - - - - - 418

Borden's Condensed Milk, - - - - - - - - - - - - - - 421

Respiratory Muscle, - - - - - - - - - - - - - - - - 424

Medication of the Respiratory Passages, - - - - - - - - - - - 426

Rupture of the Gall-Bladder, - - - - - - - - - - - - - 427

Empyema of the Left Plural Cavity treated by Injection of Iodine, - - - - - - - 429

On Bleeding from the Ear as a Consequence of Injury done to the Chin, - - - - - - 433

On Pepsin, and its Chemical and Physiological Properties, - - - - - - - - 434

### EDITORIAL DEPARTMENT.

The Opening of our Medical Schools, - - - - - - - - - - - - - 440

Professional Honor, - - - - - - - - - - - - - - - - 442

